# Novel Tick *Phlebovirus* Genotypes Lacking Evidence for Vertebrate Infections in Anatolia and Thrace, Turkey

**DOI:** 10.3390/v11080703

**Published:** 2019-08-01

**Authors:** Nergis Emanet, Sırrı Kar, Ender Dinçer, Annika Brinkmann, Sabri Hacıoğlu, Touraj Aligholipour Farzani, Zeliha Koçak Tufan, Pelin Fatoş Polat, Adem Şahan, Aykut Özkul, Andreas Nitsche, Yvonne-Marie Linton, Koray Ergünay

**Affiliations:** 1 Faculty of Medicine, Department of Medical Microbiology, Hacettepe University, Virology Unit, Ankara 06100, Turkey; 2 Department of Biology, Namık Kemal University, Tekirdağ 33110, Turkey; 3 Department of Microbiology and Immunology, Galveston National Laboratory, University of Texas Medical Branch, Galveston, GX 77555, USA; 4 Mersin University, Advanced Technology Education, Research and Application Center, Mersin 33110, Turkey; 5 Robert Koch Institute, Center for Biological Threats and Special Pathogens 1 (ZBS-1), 13353, 13352 Berlin, Germany; 6 Faculty of Veterinary Medicine, Department of Virology, Ankara University, Ankara 06110, Turkey; 7 Faculty of Medicine, Department of Infectious Diseases and Clinical Microbiology, Yıldırım Beyazıt University, Ankara 06800, Turkey; 8 Faculty of Veterinary Medicine, Department of Internal Medicine, Harran University, ŞanlIurfa 63200, Turkey; 9 Biotechnology Institute, Ankara University, Ankara 06560, Turkey; 10 Department of Entomology, National Museum of Natural History, Smithsonian Institution, Washington, DC 20560, USA; 11 Walter Reed Biosystematics Unit, Smithsonian Institution Museum Support Center, Suitland, MD 20746, USA

**Keywords:** tick, phlebovirus, species, genotype, bunyavirus, Turkey

## Abstract

We screened ticks and human clinical specimens to detect and characterize tick phleboviruses and pathogenicity in vertebrates. Ticks were collected at locations in Istanbul (Northwest Anatolia, Thrace), Edirne, Kırklareli, and Tekirdağ (Thrace), Mersin (Mediterranean Anatolia), Adiyaman and Şanlıurfa (Southeastern Anatolia) provinces from 2013–2018 and were analyzed following morphological identification and pooling. Specimens from individuals with febrile disease or meningoencephalitic symptoms of an unknown etiology were also evaluated. The pools were screened via generic tick phlebovirus amplification assays and products were sequenced. Selected pools were used for cell culture and suckling mice inoculations and next generation sequencing (NGS). A total of 7492 ticks were screened in 609 pools where 4.2% were positive. A phylogenetic sequence clustering according to tick species was observed. No human samples were positive. NGS provided near-complete viral replicase coding sequences in three pools. A comprehensive analysis revealed three distinct, monophyletic virus genotypes, comprised of previously-described viruses from Anatolia and the Balkans, with unique fingerprints in conserved amino acid motifs in viral replicase. A novel tick phlebovirus group was discovered circulating in the Balkans and Turkey, with at least three genotypes or species. No evidence for replication in vertebrates or infections in clinical cases could be demonstrated.

## 1. Introduction

Viruses classified in the genus *Phlebovirus* (order *Bunyavirales,* family *Phenuiviridae*) include many strains with a significant impact on human and animal health [1]. Pathogenic phleboviruses are frequently transmitted to susceptible vertebrates by a wide range of arthropod vectors, including phlebotomine sandflies, mosquitoes, or ticks. The mosquito-borne Rift Valley fever virus is a serious pathogen of humans and ruminants, while the sandfly-borne sandfly fever Sicilian virus and Toscana virus (TOSV) cause severe febrile diseases and central nervous system infections, respectively, in endemic regions [2]. *Phlebovirus* virions are enveloped, spherical particles with a diameter of about 100 nm. They exclusively replicate in the host cell cytoplasm and have a tripartite, single-stranded RNA genome [1]. The large (L) segment encodes for the viral RNA-dependent RNA polymerase (RdRP) and the medium (M) segment encodes for the envelope glycoproteins GN and GC, both in a negative-sense orientation [3]. The small (S) segment of the viral genome demonstrates an ambisense coding strategy and encodes for the viral nucleocapsid and a non-structural protein (NSs). Mosquito and sandfly-borne phleboviruses possess another non-structural protein (NSm), encoded in a positive-sense orientation by the M segment, which is lacking in tick-borne phleboviruses [1,3].

Tick-borne phleboviruses have largely been neglected as causative agents of human disease, until the emergence of severe fever with the thrombocytopenia syndrome virus (SFTSV) in China and the Heartland virus (HRTV) in the United States [4]. However, despite ongoing circulation and symptomatic and potentially fatal infections being reported annually, these viruses have remained the sole representatives of tick-borne phleboviruses that have been determined as highly pathogenic to man. According to current taxonomic updates on the order *Bunyavirales*, six tick-associated phlebovirus species have been recognized: Heartland banyangvirus, Huangpi kabutovirus, Kabuto mountain kabutovirus, Frijoles phlebovirus, Mukawa phlebovirus, and Uukuniemi phlebovirus [5], but a considerable number of tick-borne phleboviruses still remain unclassified. With the exception of SFTSV and HRTV [4,6,7], the epidemiology and distribution of these viruses are poorly studied and evidence for human pathogenicity of tick-borne phleboviruses is scarce. Despite the detection of particular strains in symptomatic infections and documented human or animal exposures, no outbreaks or case clusters of tick-borne phleboviruses have thus far been reported [8,9].

Increased surveillance and the availability of generic detection and unbiased sequencing techniques have accelerated novel virus discovery in ticks [10,11,12]. Several new strains have been identified during the last few years, with a rapid expansion of the list on tick-associated phleboviruses [13,14,15,16,17]. We previously reported preliminary findings indicating the activity of indigenous tick phleboviruses in Anatolia, Turkey [11,18]. This study was carried out to determine the prevalence and diversity of phleboviruses circulating in Anatolia, as well as in new regions across Turkey with a high population density, and to assess clinical impact and vertebrate pathogenicity of these strains.

## 2. Materials and Methods

### 2.1. Ethics Approvals

The removal of ticks from infested domestic animals was performed with the informed consent and cooperation of the caretakers or owners. Stored human specimens were included for testing with approvals from relevant local or institutional ethics committees (Hacettepe University non-interventional clinical research ethics board, FON.12/05-5, 2014; Ankara Training and Research Hospital ethics board, 13.07.11/0426). Animal experiments were carried out according to national regulations on the operation and procedure of animal experiments’ ethics committees (regulation Nr. 26220, Date: 9 July 2006) and approved by the Ankara University local animal ethics board (Nr. 2019-6-61, Date: 6 March 2019). For field-collected questing ticks and those collected from domesticated animals, no local or regional ethics committee approval was required.

### 2.2. Tick Specimens 

**Figure 1:**
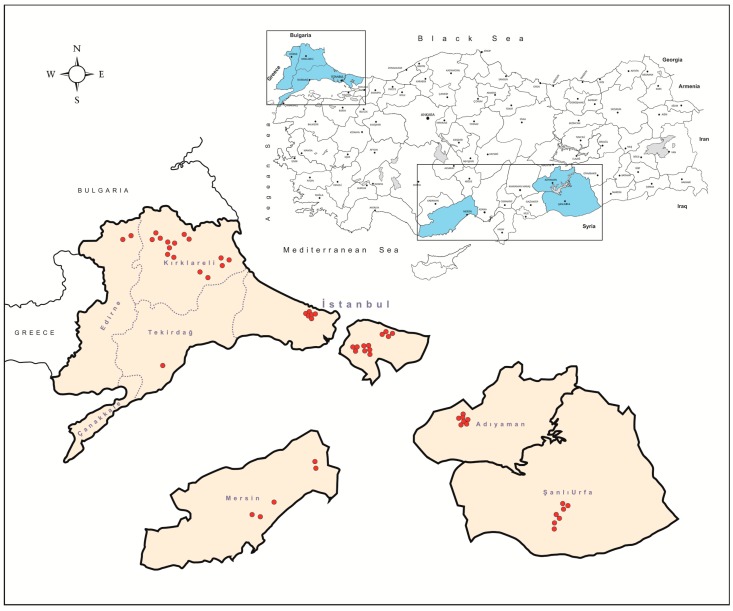
Illustrative map of the sampling locations in the study.

The tick specimens were collected at 37 locations in Istanbul (Northwest Anatolia, Thrace region), Edirne, Kırklareli, and Tekirdağ (Thrace region), Mersin (Southern Anatolia, Mediterranean region), Adıyaman and Şanlıurfa (Southeastern Anatolia) provinces, between 2013–2018 (Figure 1). Collections in the Istanbul province was carried out by flagging, using a 75 × 100 cm cloth, over both low and high vegetation. All other ticks were collected directly from infested cattle (*Bos taurus*), sheep (*Ovis aries*), goats (*Capra aegagrus hircus*), and dogs (*Canis familiaris*), at privately-owned farms or animal shelters. All specimens were kept in separate vials, transferred to the laboratory in dry ice and identified morphologically to the species level using appropriate taxonomic keys [19,20,21,22,23]. Subsequently, the specimens were pooled according to the collection site, species, and developmental stage of up to a maximum of 50 individuals per pool and stored at −80 °C for further analyses.

### 2.3. Specimen Processing

Tick pools were disrupted by vortexing with 4.5 or 7.0 mm tungsten carbide beads (QIAgen, Hilden, Germany) in 500–700 μL of Eagle’s minimal essential medium, supplemented with 5% fetal bovine serum and 1% l-glutamine. Subsequently, the ground pools were clarified by centrifugation at 4000 rpm for 4 min, and the supernatant from each pool was aliquoted and stored at −80 °C. Nucleic acid extraction from tick pool supernatants was carried out using the High Pure Viral Nucleic Acid Kit (Roche Diagnostics, Mannheim, Germany), and complementary DNA synthesis with random hexamers carried out using the High-Capacity cDNA Reverse Transcription Kit (Thermo Fisher Scientific, Hennigsdorf, Germany), as directed by the manufacturers.

### 2.4. Virus Screening

Processed tick pools were screened by generic polymerase chain reaction (PCR) assays. Two PCR assays developed for the optimized detection of tick-borne phleboviruses were employed [10]. The assays utilized generic primer sets (ppL1 and ppL2) which target the well-conserved viral polymerase functional motifs, premotif A and motif B [10]. The primer sets demonstrated detection limits of 10^2^–10^3^ TCID_50_ equivalents for several tick-borne phleboviruses, using viral RNA from culture supernatants [10]. The vero cell-grown Toscana virus isolate ISS.Phl.3, a sandfly-borne phlebovirus that can be robustly detected via both primer sets, was used for optimization and as a positive control.

Amplified products of the screening assays were visualized using a ChemiDoc XRS+ imaging system (Bio-Rad Laboratories, Munich, Germany) via ethidium bromide staining following electrophoresis in 1.5% agarose gels. 

### 2.5. Human Specimens 

To identify probable human infections with tick-associated phleboviruses, we previously tested stored sera from individuals with the laboratory diagnosis of Crimean–Congo hemorrhagic fever (CCHF), cerebrospinal fluid (CSF), and/or sera from individuals with febrile disease or meningoencephalitic symptoms of an unknown etiology. The specimens were stored at −80 °C and available as serum, purified RNA, or cDNA, processed using the High Pure Viral Nucleic Acid Kit (Roche Diagnostics, Mannheim, Germany) and RevertAid First Strand cDNA Synthesis Kit (Thermo Fisher Scientific). Nucleic acid purification and cDNA synthesis in serum specimens were carried out as described for tick pools. All specimens were screened for phleboviruses using the generic primer sets ppL1 and ppL2, as described above.

### 2.6. Virus Isolation

Available aliquots of the phlebovirus positive tick pools were inoculated onto semi-confluent monolayers of African green monkey kidney (Vero E6) cells, obtained from the cell culture collection of the Department of Virology, Faculty of Veterinary Medicine, Ankara University. Approximately 400 μL of pool homogenates were filtered through a 0.22-μm sterile membrane filter (Merck Millipore, Darmstadt, Germany), diluted in equal volumes of Dulbecco’s modified Eagle’s medium (DMEM), and inoculated onto Vero cells in T25 flasks (Nunc, Roskilde, Denmark). Following adsorption to the cells for an hour, 5 mL of DMEM, supplemented with 5% of fetal bovine serum, l-glutamine, 100 U/mL penicillin, and 100 μg/mL streptomycin, were added. The cells were incubated at 37 °C with 5% CO_2_ and monitored daily for cytopathic effects. Blind passages were performed weekly up to the fourth passage and culture supernatants were tested for viral nucleic acids via the screening assays. 

Selected tick pool homogenates were further used for the intracerebral inoculation of suckling mice. Following filtration through a 0.22-μm sterile membrane filter (Merck Millipore, Darmstadt, Germany) and inoculation of approximately 20 μL of the homogenate, the mice were observed twice daily for clinical signs. Following observation, the mice were euthanized by CO_2_ exposure and cervical dislocation and brain, liver, spleen, kidney tissues, and sera were harvested. The tissues were processed and tested for phleboviruses as described above.

### 2.7. Sanger and Next Generation Sequencing (NGS)

Products of the expected size amplified via the screening assays were characterized by sequencing. Following clean-up up using the PureLink PCR Purification Kit (Thermo Fisher Scientific, Hennigsdorf, Germany), the products were sequenced using forward and reverse PCR primers and the BigDye Terminator v3.1 Cycle Sequencing Kit (Thermo Fisher Scientific, Hennigsdorf, Germany), in an ABI PRISM 3500xL Dx Genetic Analyzer (Thermo Fisher Scientific, Hennigsdorf, Germany). 100 μL aliquot of the selected tick pools were included in NGS. Ambion DNase I and RNase Cocktail (Thermo Fisher Scientific, Hennigsdorf, Germany) were used for the initial treatment of the specimens, according to the manufacturer’s protocols. Subsequently, unencapsidated nucleic acids were removed using the Agencourt AMPure XP purification system (Beckman Coulter Biosciences, Krefeld, Germany). The QIAamp Viral RNA Mini Kit (Qiagen, Hilden, Germany) was employed for purification and 5 ng of RNA was reverse transcribed into double-stranded cDNA using random hexamers via SuperScript IV Reverse Transcriptase (Thermo Fisher Scientific, Hennigsdorf, Germany) and the NEBNext mRNA Second Strand Synthesis Module (New England Biolabs, Frankfurt am Main, Germany). The cDNA was further cleaned up using the Agencourt AMPure XP reagent (Beckman Coulter Biosciences, Krefeld, Germany) standard protocol and total yield and size distribution were checked via the Agilent 2100 Bioanalyzer (Agilent Technologies, Waldbronn, Germany). Fragmentation, adaptor ligation, and amplification steps were performed using the Nextera XT DNA Library Prep kit (Illumina, San Diego, CA, USA), according to the manufacturer’s recommendations. The sequencing run was carried out using the Illumina MiSeq 1500 (Illumina, San Diego, CA, USA) in the paired-end mode.

### 2.8. Sequence Data Analysis

Sequences obtained by Sanger sequencing were analyzed using Geneious software v11.1.5 (Biomatters Ltd, Auckland, New Zealand). NGS raw data were de-multiplexed and extracted in a fastq format. Adaptor removal and trimming for quality and length (phred score of 33 and 30 base pairs (bp) minimum length) were carried out using Trimmomatic v0.35 [24]. Acquired reads were aligned to an in-house curated database, comprising phlebovirus sequences deposited in the GenBank, using MALT (MEGAN alignment tool, v0.3.8) and MEGAN (Metagenome Analyzer, v.6.12.3) [25,26]. Aligned reads were extracted and assembled into contigs using Velvet (v.1.2.10) [27]. The contigs were mapped to closely related virus sequences, checked for heterogeneity by visual inspection, and pairwise identity values via Geneious software.

BLASTn, BLASTn optimized for highly similar sequences (MEGABLAST) and BLASTp algorithms were used for nucleotide and deduced amino acid similarity searches in the public databases [28]. Nucleotide and putative amino acid alignments and pairwise sequence comparisons were generated using CLUSTALW [29]. Screening for recombination among related tick phleboviruses was undertaken using algorithms implemented in the RDP4 software [30], in the default settings. SimPlot v.3.5.1 was used for generating nucleotide similarity plots and recombination analysis using Bootscan [31]. Evolutionary history was inferred via the maximum-likelihood method based on the model estimated as the optimal substitution model individually for each alignment according to the Bayesian information criterion and conducted using MEGAX [32]. Conserved protein domain and motif searches were performed in the PFAM database [33,34].

## 3. Results

### 3.1. The Tick Cohort and Screening Findings

We processed a total of 7492 ticks, originating from locations at Kırklareli (3682, 49.1%), Istanbul (3044, 40.6%), Tekirdağ (288, 3.8%), Mersin (192, 2.5%), Edirne (140, 1.8%), Şanlıurfa (78, 1%), and Adıyaman (68, 0.9%). The majority of the specimens comprised of adult ticks (female: 2623, 35%; male: 1826, 24.4%), followed by larvae (2094, 27.9%), and nymphs (949, 12.6%). The most abundant group tested was *Ixodes* sp. larvae (2212, 29.5%), followed by *Hyalomma marginatum* (1022, 13.6%), *Hyalomma scupense* (962, 12.8%), *Haemaphysalis parva* (673, 8.9%), and *Rhipicephalus turanicus* (610, 8.1%). The complete list and distribution of tick species according to the developmental stage and collection site are provided in Table S1. 

The tick specimens were screened in 609 pools and 26 pools (4.2%) were positive by the generic tick phlebovirus PCRs (Table 1). These positive pools originated from the Kırklareli province which borders Bulgaria (53.8% (14/26); *Haemaphysalis punctata*, *H. scupense*, *Rhipicephalus bursa*, *R. turanicus*) and Tekirdağ province (19.2% (5/26); *R. turanicus*) in Northwest Turkey, the Mediterrenean province of Mersin (19.2% (5/26); *R. bursa*, *Rhipicephalus sanguineus* sensu lato), and the southern province of Şanlıurfa (7.6% (2/26); *R. sanguineus* s.l.), which borders Syria, indicating widespread coverage across the country (Figure 1, Table 1). Pools with detectable virus sequences comprised of female and male adults of *R. turanicus* (11/26, 42.3%), *R. bursa* (6/26, 23.1%), *R. sanguineus* s.l. (5/26, 19.2%), *Hae. punctata* (2/26, 7.6%), and *H. scupense* (2/26, 7.6%) (Table S2). Pools with larvae or nymphs remained negative. Virus infection rates according to location and tick species were further assessed, revealing varying rates of 0.2 to 3.1% (Table 1).

**Figure 2:**
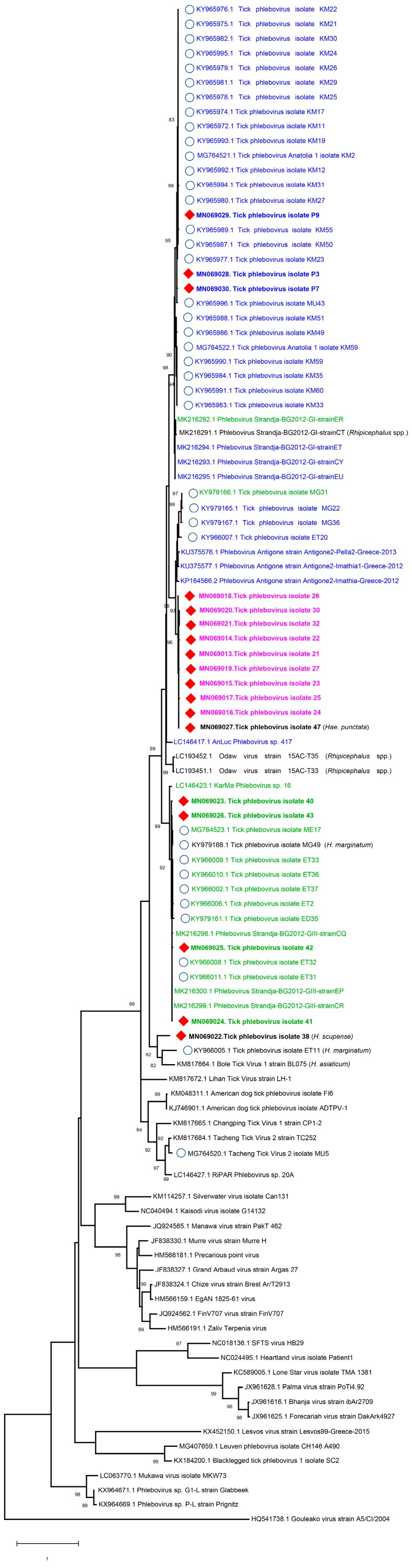
The maximum likelihood analysis of the partial tick phlebovirus L segment sequences (412 bp). The tree is constructed using the General Time Reversible (GTR) model, Gamma distributed with Invariant sites (G+I) for 500 replications. The sequences characterized in this study are given in bold and indicated with a symbol (red diamond), GenBank accession number, and pool code. Global virus strains are indicated by GenBank accession number and strain/isolate name. Viruses previously characterized in Turkey are indicated with blue circles. Color codes indicating tick hosts are provided for prominent virus clades (blue: *R. sanguineus* s.l., green: *R. bursa*; and pink: *R. turanicus*). Other tick species are given in parantheses. Bootstrap values higher than 80 are provided. The mosquito-borne phlebovirus Gouleako is included as an outgroup.

Amplicon sequencing in PCR-positive tick pools provided 508–539 bp segments of the virus polymerase. The sequences showed 0.4%–19.9% nucleotide diversity and up to 91% identity to previously-characterized near-complete tick phlebovirus L segment sequences from Anatolia. In the maximum likelihood analysis, a differential clustering of the sequences was observed (Figure 2). The clustering pattern was unrelated to geographical origin, but appeared species-dependent to a certain extent, as sequences from *R. bursa, R. sanguineus* s.l., and *R. turanicus* were placed in distinct groups. This grouping also included virus sequences previously characterized from geographically segregated regions in Anatolia, as well as those recently reported from the Strandja region in Bulgaria [11,17,18], which is close to the sampling sites in Kırklareli. These findings suggest differential adaptation processes in various species of ticks. 

### 3.2. Human Findings

A total of 59 previously-collected clinical specimens (47 sera and 12 CSF) from 47 individuals were screened for phleboviruses. The study cohort included 10 serum specimens from CCHF virus-infected individuals with viral loads of 2.14 × 10^5^–2.36 × 10^8^ genome copies and detectable IgM-IgG antibodies in five samples. Moreover, 11 sera from individuals with a febrile disease of an unknown etiology (no tick bites) and 14 sera from individuals with a history of tick bites were included. Finally, serum-CSF pairs from 12 patients with acute onset central nervous system infections without an identifiable etiology were evaluated. All specimens were negative in generic tick phlebovirus assays in repeated tests.

### 3.3. Virus Isolation

All available tick homogenates were used for Vero cell inoculations. However, no cytopathic effects were observed during four consecutive blind passages and culture supernatants remained negative in screening assays. Available aliquots from two tick pools were used for intracerebral inoculation of suckling mice. During the observation period of 0–14 days, the mice did not exhibit any abnormalities or symptoms. They were sacrificed on day 14 and tissue specimens were obtained aseptically. No gross pathology was visible during necropsy. Aseptically-obtained sera and tissue homogenates from the brain, liver, spleen, and kidney were negative in screening PCRs. 

### 3.4. NGS Findings and Genomic Characterization of L Segments

**Figure 3:**
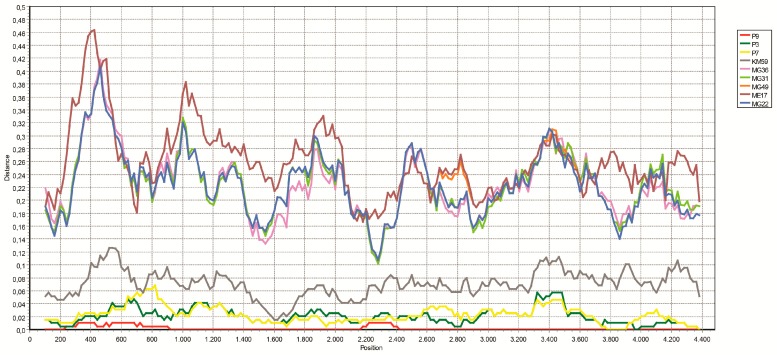
Plots of similarity of the near-complete polymerase coding alignment (4480 bp) of tick phlebovirus Anatolia 1 isolate KM2 (MG764521) with closely-related strains (GapStrip: On, Reps: 1000, Maximum Likelihood, T/t: 2.0). The curves indicate comparisons between the target and reference genomes (P9: MN069029, P3: MN069028, P7: MN069030, KM59: MG764522, MG36: KY979167, MG31: KY979166, MG49: KY979168, ME17: MG764523, MG22: KY979165). Each point plotted is the percent identity within a sliding window 200 bp, wide centered on the position plotted, with a step size between points of 20 bp.

We employed NGS directly on three pools comprising of *R. sanguineus* s.l. specimens with detectable tick phlebovirus partial sequences. We further utilized three additional pools of *R. bursa*, *R. sanguineus* s.l., and *R. turanicus*, negative in screening, as controls (Table S2). The pools provided total reads within the range of 0.9–1.5 × 10^6^ and tick phlebovirus sequences comprising of 0.3%–3.1% of the total reads were identified in pools positive in screening. In these pools, 6205, 6209, and 6396 bp contigs, covering a near-complete coding region of the phlebovirus L genomic segment, were assembled. BLASTn searches revealed the highest identities (79.1%–99.8% in pairwise comparisons) to tick-associated phleboviruses previously characterized in Turkey. These viruses were mainly detected in Mediterranean and Aegean regions of Anatolia and suggested to constitute a novel tick-associated phlebovirus, tentatively named as tick phlebovirus Anatolia [11,18]. The SimPlot analysis revealed the current sequences (P3, P7, and P9) to be most closely-related to the prototype tick phlebovirus Anatolia sequences KM2 and KM59 (Figure 3). 

**Figure 4:**
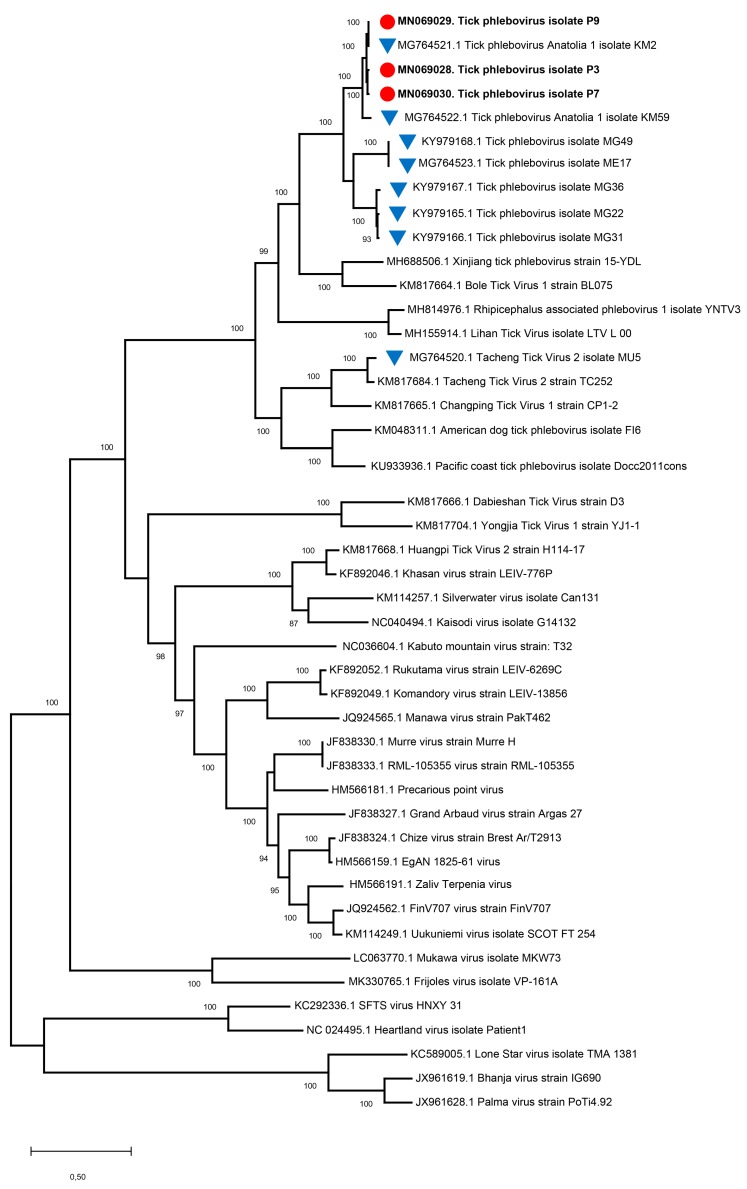
The maximum likelihood analysis of the L segment near-complete coding sequences of tick phleboviruses (4642 bp). The tree is constructed using the General Time Reversible (GTR) model, Gamma distributed with Invariant sites (G+I) for 500 replications. The sequences characterized in this study are given in bold and indicated with a symbol (red circle), GenBank accession number, and pool code. Global virus strains are indicated by the GenBank accession number and strain/isolate name. Viruses previously characterized in Turkey are indicated with a blue triangle. Bootstrap values higher than 80 are provided.

This is further supported by the maximum likelihood tree, where three distinct, monophyletic virus clades can be distinguished among tick-associated phleboviruses (Figure 4). One clade is formed by the current sequences and KM2/KM59, the others comprise of MG49/ME17 and MG36/MG22/MG31 viruses, respectively. Follow-up analyses based on the relatively shorter regions to include related viruses from Bulgaria and Greece also revealed comparable tree topologies and a close relation of the Antigone, Strandja-GI, and Strandja-GIII viruses to these clades (Figures S3 and S4). No evidence of recombination could be detected among the sequences, using manual Geneconv, Bootscan, MaxChi, Chimaera, SiScan, 3Seq tools, run in default settings in RDP4. Bootscan analysis performed in SimPlot also failed to recognize any recombination events.

### 3.5. Analysis of Putative Coding Regions

The characterized L segment sequences in tick pools covered the viral RNA-dependent RNA polymerase open reading frame (ORF) and were subsequently translated in silico to putative polyproteins of 2068, 2069, and 2132 amino acids, respectively. Several Bunyaviruses motifs were detected, including conserved domains of RNA-dependent RNA polymerase (pfam04196, superfamily cl20265), the N-terminus endonuclease domain (pfam15518), and a viral protein of an unknown function (DUF3770) (pfam12603).

**Figure 5:**
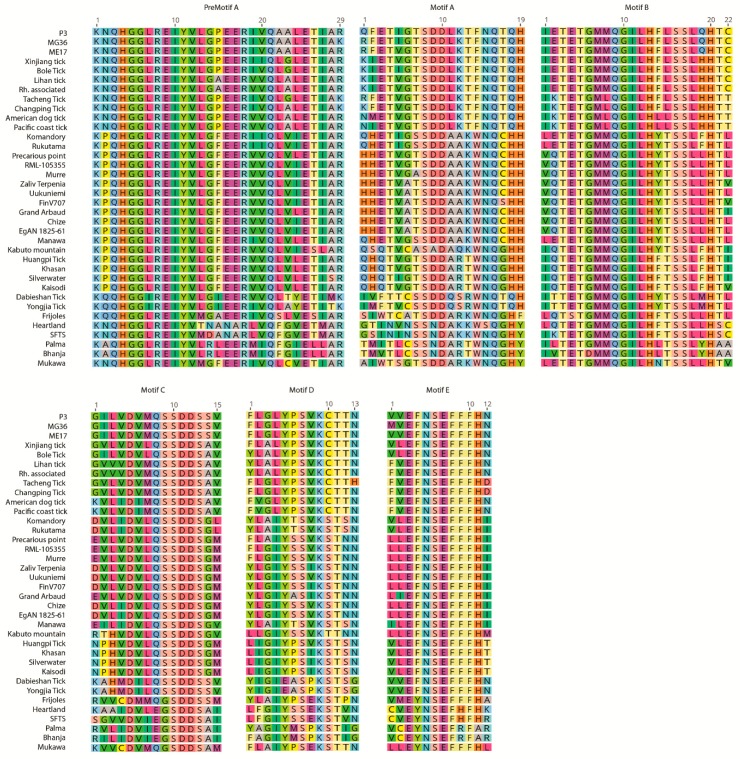
Alignment of the phlebovirus RNA-dependent RNA polymerase conserved motifs, as located on the amino acid residues 936–970 (premotif A), 1008–1026 (motif A), 1107–1129 (motif B), 1152–1168 (motif C), and 1197–1223 (motif D/E) positions on the Uukuniemi virus genome L segment (NP941973). GenBank accession and sequence information on individual strains used in comparison are provided in Figure 4.

We further analyzed previously-described RNA-dependent RNA polymerase regions identified in phlebovirus genomes, including tick-associated strains [35]. Region I and II are located in the amino terminus of the enzyme and conserved in arena and bunyaviruses [36]. Centrally-located region III is present in all RNA viruses with RNA polymerase-based replication and contains the polymerase motifs preA, A to E [37]. Additional conserved regions (IV to VI) have also been described, and likely to participate in polymerase functions [38]. We aligned and deduced polyproteins obtained from Anatolia and observed identical polymerase motifs B–D and region V. The remaining motifs and regions were significantly conserved, with few amino acid substitutions consistent with phylogenetic clades. Then, we selected representative sequences from each clade (P3, MG35, and ME17) and aligned these with other tick-associated phleboviruses. Overall, unique amino acid patterns were observed in sequences from Anatolia for regions I–VI and in region III specific motifs, compared to major tick phleboviruses (Figure 5, Figure S5). Related viruses were noted as the Xinjiang tick phlebovirus, Bole tick Virus 1, *Rhipicephalus*-associated phlebovirus 1, and Lihan tick virus, with 80%–93.1% identities in region III motifs and 55.8%–88% identities in the remaining regions. 

## 4. Discussion

In an attempt to detect and characterize tick-borne phleboviruses, we screened ticks and human clinical specimens using generic amplification and NGS in this study. The tick cohort encompassed a high number of specimens (*n* = 7492) from seven provinces across Turkey over five years (2013–2018), various developmental stages of ticks (larvae to adults), and regions with diverse ecological niches and fauna (Figure 1).

Phlebovirus nucleic acids were detected in 4.2% of 609 pools. Positive pools originated from Thrace (Kırklareli and Tekirdağ provinces), Mediterranean Anatolia (Mersin province), and Southeastern Anatolia (Şanlıurfa province) (Table 1). Viral sequences were detected in *Rhipicephalus* (*bursa*, *sanguineus* s.l., and *turanicus*) spp., *Hae. punctate*, and *H. scupense*, while *Ixodes* spp., despite being the most frequent species in particular locations, remained negative (Table 1, Table S1). Analysis of the partial viral L segment sequences obtained by sequencing revealed considerable diversity (up to 19.9% in pairwise comparisons). Furthermore, maximum likelihood analysis suggested a large-scale clustering of the sequences according to tick species (Figure 2). However, several exceptions, such as the sequences identified in *Hae. punctata* and *H. marginatum*, as well as host inconsistencies with previous reports were recognized. Geographical segregation was not detected, as previously-identified and current sequences were grouped together, regardless of the collection site. Similar findings were also reported from Europe, where closely related sequences, likely to represent a single phlebovirus species were detected in the same tick species or genera, suggesting some sort of host specificity [17]. In countries with available data, various phlebovirus clades seem to coexist in ticks. Moreover, distinct phlebovirus clades originating from Bulgaria, Greece, Turkey, or Portugal were phylogenetically-related and detected in particular species of ticks [11,14,15,17,18,39]. We previously reported on preliminary evidence for the circulation of several distinct phlebovirus clades, collected in specimens from the Aegean and Mediterranean, as well as Central, Eastern, and Southeastern Anatolia [18]. Our current findings further confirm these observations and document tick phleboviruses in the Thrace region as well. A recent analysis, which excludes sequences from Turkey and Bulgaria, indicated the Middle East as the most likely origin of these viruses, with evidence for various routes for introduction and dispersion into Europe [39]. Interestingly, despite repeated detections in *Rhipicephalus* and *Haemaphysalis* spp., we have yet to identify tick phleboviruses in Turkish *Ixodes* and *Dermacentor* ticks, despite reports elsewhere in Europe [13,14,40]. A similar observation was noted in a survey from the Iberian peninsula as well [39], suggesting differential susceptibility among tick genera. The host range of tick phleboviruses and the ability to infect multiple species remains to be elucidated.

In our cohort, we detected tick phlebovirus nucleic acids exclusively in adult ticks, comprised of female and male specimens, despite an abundance of nymphs and larvae (Table S1). We previously observed infected pools comprised of both adults and nymphs, without detailed characterization of the infected specimens [18]. Viral sequences could be amplified from tick eggs, nymphs, and adults in previous reports, and a vertical transmission route likely to support virus maintenance was deemed possible [17,39]. The lack of nymph/larva specimens with detectable virus sequences in this study may represent a sampling bias, as few nymphs/larvae of the frequently-infected species were available. Transmission routes and maintenance mechanisms also await to be explored deeply for a better understanding of tick phlebovirus life cycle in nature.

In this study, we could obtain near-complete coding sequences of three putative new phleboviruses and were able to carry out a comprehensive analysis of the RNA-dependent RNA polymerase regions. Phylogenetic analyses performed on L segment genome fragments of various sizes revealed that the sequences represent three distinct clades or genotypes and included some of the previously-described viruses from Bulgaria and Greece (Figure 4, Figures S3 and S4). This is further supported by deduced amino acid alignments of the conserved regions and motifs of the enzyme, where unique fingerprints could be identified for each clade (Figure 5, Figure S5). Demonstrating a greater diversity than mosquito or sandfly-borne phleboviruses, several tentative tick phlebovirus groups, based on genetic and serological relationships, have been proposed, namely: The SFTSV, Bhanja virus, Uukuniemi virus, and Kaisodi virus groups [12]. The tick phlebovirus group we identified, including the current sequences and those detected in various European locations, is distantly related to these groups and shares a common ancestor with several viruses from Asia and America (Pacific coast tick phlebovirus, American dog tick phlebovirus, Changping tick virus 1, Tacheng tick virus 2, Lihan tick virus) (Figure 5). Phylogenetically, the group is related with the Xinjiang tick virus and Bole tick virus 1 strains. Given the available evidence, it is likely to represent a novel tick phlebovirus species with genotypes formed by local sequences detected in Anatolia and the Balkans. Individual genotypes may have adapted to different tick genera or species, as suggested by the partial sequence data. Interestingly, they also seem to coexist in particular locations, in addition to genetically-distant Tacheng tick virus 2 (strain MU5) and Strandja-GII phlebovirus in Turkey and Bulgaria, respectively (Figure 5). These findings indicate that several closely- and distantly-related tick phleboviruses cocirculate in given regions, with unknown consequences. 

A major hurdle in characterizing the genome of this novel group and determining precise genetic relationships with other tick phleboviruses is the availability of relatively limited sequence information. We could only characterize the viral L segment in this study, although S segment sequences were obtained in our previous efforts [11,18]. Strikingly, several surveys involving the metagenomic investigation of field-collected ticks reported similar findings, where no information on the M-segment of the putative tick phleboviruses, encoding for the envelope glycoprotein precursors, could be retrieved [13,41,42,43]. Moreover, these viruses all share common ancestors with the currently-described genotypes from Turkey (Figure 5). The lack of M-segment sequences may be due to a significant divergence, hampering identification in NGS [43]. These strains may also completely lack the M-segment and employ other mechanisms for cellular entry. It is also possible for some to be endosymbionts and use transovarial or alternate routes for transmission [41]. The lack of culture-adapted isolates is another obstacle for detailed biological and genomic characterization. So far, none of the viruses described in various regions in Europe could be isolated using vertebrate cell lines, despite some evidence for transient viral maintenance in tick cells [14,17]. Our attempts for virus isolation in Vero cells or suckling mice also failed in this study. It remains to be determined whether these viruses can produce infectious virions, may replicate or be rescued by co-infecting helper viruses, or constitute endosymbiotic relatives of the tick phleboviruses. Nevertheless, some of the recently-described tick phleboviruses such as Mukawa, Kabuto mountain, and Guertu viruses, distantly-related to the current groups, could be successfully propagated in various cell lines [12,44,45]. They can further adapt to, and induce pathological changes in, mice, indicating that some of the novel tick phleboviruses have the potential to emerge as human or animal pathogens.

Among the currently-known tick phleboviruses, only SFTSV and HRTV are well-established as human pathogens. Symptomatic individuals infected with these viruses exhibit fever, fatigue, diarrhea, thrombocytopenia, and leukopenia, as well as elevated levels of liver-associated transferases [6,7,46]. Moreover, neurological symptoms and encephalitis have been reported in some SFTSV infections [46,47,48]. These viruses can also infect various animals including cattle, sheep, pigs, dogs, goats, and chickens [6,7]. However, only a few tick phleboviruses have been implicated in disease in Europe. Bhanja virus is mainly pathogenic for livestock, but reported to cause human febrile disease following natural and laboratory infections [9,49,50]. Exposure to the Uukuniemi virus was also documented in cattle from Finland [8]. We did not identify tick phlebovirus RNAs in clinical specimens from CCHFV cases or individuals with febrile and/or neurological disease or with an unknown etiology in this study. However, the symptoms in SFTSV and HRTV infections, especially in mild cases, are generally non-specific and can easily be misdiagnosed as tick-borne bacterial diseases without proper laboratory testing [7]. Therefore, the identification of isolated human infections or outbreaks require the inclusion of tick phleboviruses in the diagnostic work-up of cases with compatible clinical presentation. For this purpose, previously published generic amplification methods appear adequate.

Finally, the potential of tick phleboviruses as emerging human or animal pathogens must be elaborated. The findings of this study as well as previous reports have so far failed to demonstrate vertebrate pathogenicity of any of the tick phleboviruses detected in Europe. However, recent experiments utilizing virus-like particles have shown that reassortment events occur between pathogenic and non-pathogenic tick phleboviruses and viral glycoproteins can package genomes of related phleboviruses [51]. Therefore, coinfections in ticks or reservoir animals may lead to the generation of viable reassortant progenies and the emergence of a new pathogenic strain. This is especially important in regions such as China and Japan, where the SFTSV and other phleboviruses cocirculate in various tick species. 

In conclusion, we described a novel tick phlebovirus group with at least three genotypes or species, circulating in questing, and host removed ticks from Thrace and Anatolia. No evidence for replication in vertebrates or infections in clinical cases could be demonstrated.

## Figures and Tables

**Table 1:** Distribution of tick pools with detectable phleboviruses. The infection rate was calculated assuming a single infected specimen in each pool. All positive pools includ adult ticks.

Province	Species	Pools (n)	Positive Pool (n/%)	Infection Rate (%)
Mersin	*R. bursa* (*n* = 65)	11	2/18.2	3.1
	*R. sanguineus* s.l. (*n* = 117)	14	3/21.4	2.6
Şanlıurfa	*R. sanguineus* s.l. (*n* = 67)	10	2/20	2.9
Tekirdağ	*R. turanicus* (*n* = 264)	12	5/41.6	1.9
Kırklareli	*R. bursa* (*n* = 438)	55	4/7.2	0.9
	*R. turanicus* (*n* = 319)	42	6/14.3	1.9
	*Hae. punctata* (*n* = 73)	15	2/13.3	2.7
	*H. scupense* (*n* = 962)	49	2/4.1	0.2

## Supplementary Materials

The following are available online at https://www.mdpi.com/1999-4915/11/8/703/s1, **Table S1:** Distribution of tick species according to collection site, sex, and developmental stage. **Table S2:** Features of the tick pools positive in screening and evaluated using NGS. **Figure S3:** The maximum likelihood analysis of the partial tick phlebovirus L segment sequences (707 bp). The tree is constructed using the General Time Reversible (GTR) model, Gamma distributed with Invariant sites (G+I) for 500 replications. The sequences characterized in this study are given in bold and indicated with a symbol (red diamond), GenBank accession number, and pool code. Global virus strains are indicated by GenBank accession number and strain/isolate name. Viruses previously characterized in Turkey are indicated with green circles. Bootstrap values higher than 80 are provided. **Figure S4:** The maximum likelihood analysis of the partial tick phlebovirus L segment sequences (347 bp). The tree is constructed using the General Time Reversible (GTR) model, Gamma distributed with Invariant sites (G+I) for 500 replications. The sequences characterized in this study are in bold with the GenBank accession number and pool code. Global virus strains are indicated by GenBank accession number and strain/isolate name. Bootstrap values higher than 80 are provided. **Figure S5:** Alignment of the phlebovirus RNA-dependent RNA polymerase conserved regions other than region III, as located on the amino acid residues 107–131 (Region I), 673–731 (Region II), 1225–1324 (Region IV), 1420–1458 (Region V), and 1551–1646 (Region VI) on the Uukuniemi virus genome L segment (NP941973). GenBank accession and sequence information on individual strains used in comparison are provided in Figure 4.
