# Supplementary figures and images for: Novel Tick Phlebovirus Genotypes Lacking Evidence for Vertebrate Infections in Anatolia and Thrace, Turkey
Source: Viruses. 2019 Aug 1;11(8):703. doi: 10.3390/v11080703 (PMC6723390; doi:10.3390/v11080703)

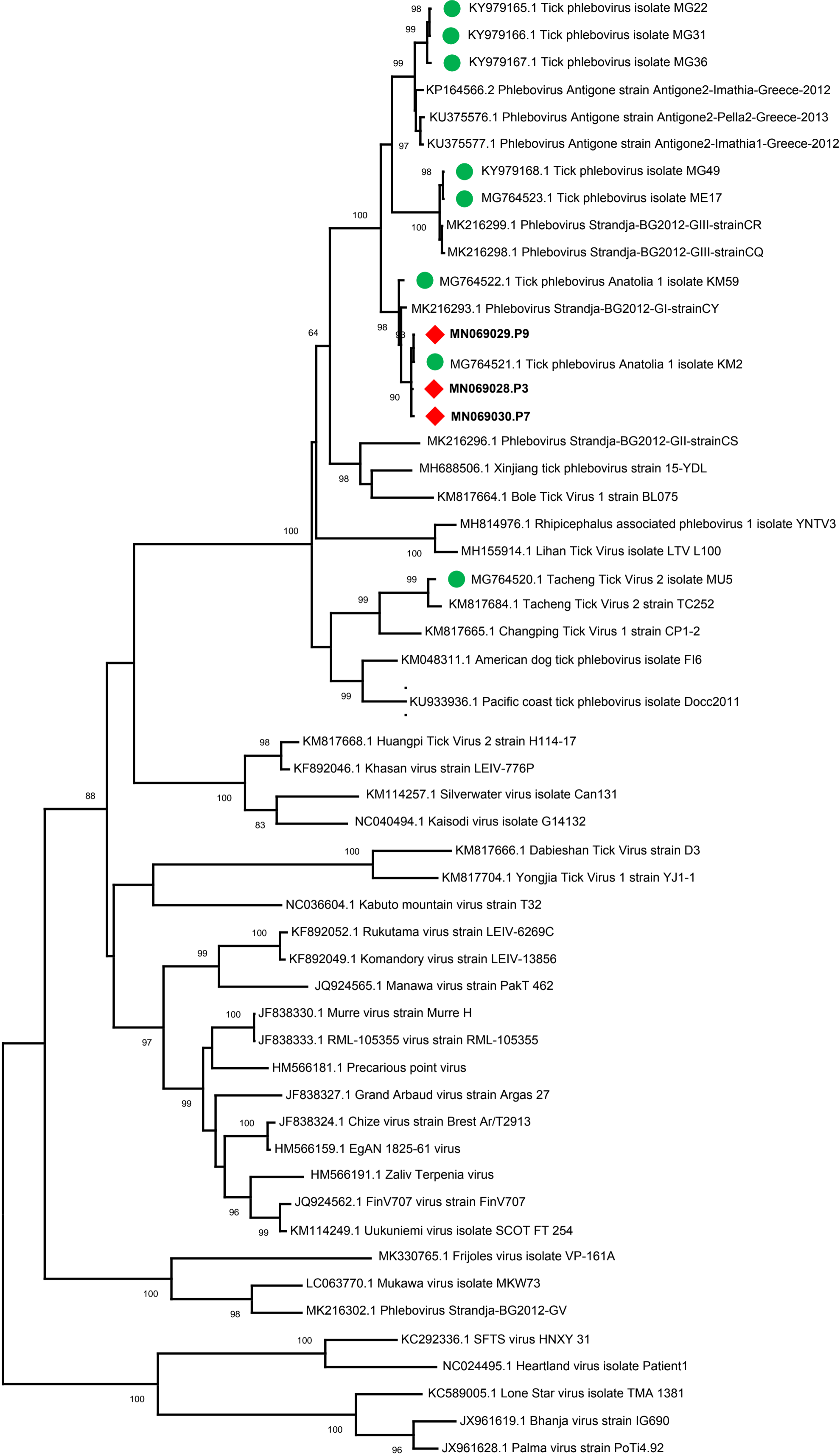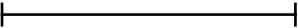

0.5

Supplement: Supplementary file 1 [file viruses-11-00703-s001.zip › FigureS3revision.pdf]
