# Supplementary material for: Novel Tick Phlebovirus Genotypes Lacking Evidence for Vertebrate Infections in Anatolia and Thrace, Turkey
Source: Viruses. 2019 Aug 1;11(8):703. doi: 10.3390/v11080703 (PMC6723390; doi:10.3390/v11080703)
Supplement: Supplementary file 1 [file FigureS4revision.pdf › FigureS4revision.pdf]

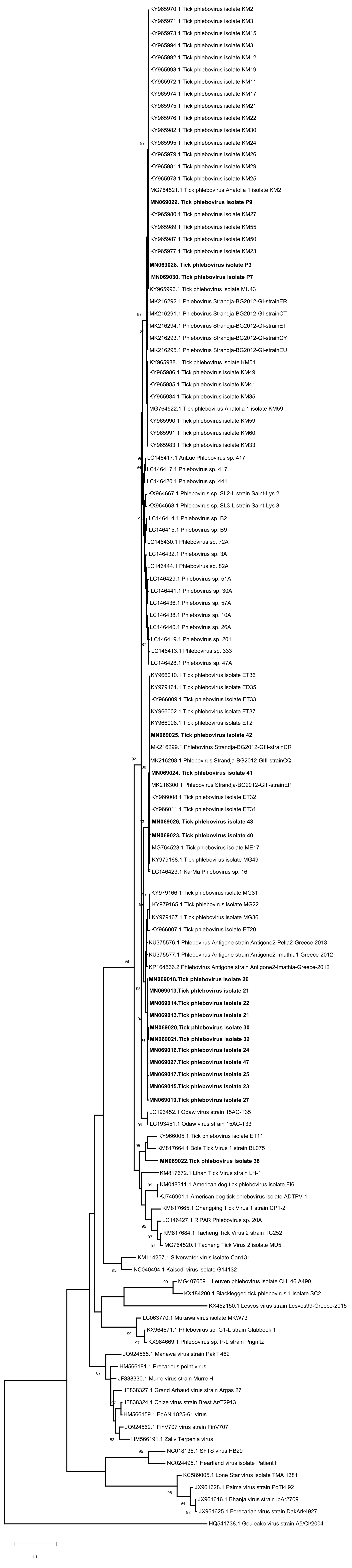
KY965970.1 Tick phlebovirus isolate KM2
KY965971.1 Tick phlebovirus isolate KM3
KY965973.1 Tick phlebovirus isolate KM15
KY965994.1 Tick phlebovirus isolate KM31
KY965992.1 Tick phlebovirus isolate KM12
KY965993.1 Tick phlebovirus isolate KM19
KY965972.1 Tick phlebovirus isolate KM11
KY965974.1 Tick phlebovirus isolate KM17
KY965975.1 Tick phlebovirus isolate KM21
KY965976.1 Tick phlebovirus isolate KM22
KY965982.1 Tick phlebovirus isolate KM30
KY965995.1 Tick phlebovirus isolate KM24
KY965979.1 Tick phlebovirus isolate KM26
KY965981.1 Tick phlebovirus isolate KM29
KY965978.1 Tick phlebovirus isolate KM25
MG764521.1 Tick phlebovirus Anatolia 1 isolate KM2
MN069029. Tick phlebovirus isolate P9
KY965980.1 Tick phlebovirus isolate KM27
KY965989.1 Tick phlebovirus isolate KM55
KY965987.1 Tick phlebovirus isolate KM50
KY965977.1 Tick phlebovirus isolate KM23
MN069028. Tick phlebovirus isolate P3
MN069030. Tick phlebovirus isolate P7
KY965996.1 Tick phlebovirus isolate MU43
MK216292.1 Phlebovirus Strandja-BG2012-GI-strainER
MK216291.1 Phlebovirus Strandja-BG2012-GI-strainCT
MK216294.1 Phlebovirus Strandja-BG2012-GI-strainET
MK216293.1 Phlebovirus Strandja-BG2012-GI-strainCY
MK216295.1 Phlebovirus Strandja-BG2012-GI-strainEU
KY965988.1 Tick phlebovirus isolate KM51
KY965986.1 Tick phlebovirus isolate KM49
KY965985.1 Tick phlebovirus isolate KM41
KY965984.1 Tick phlebovirus isolate KM35
MG764522.1 Tick phlebovirus Anatolia 1 isolate KM59
KY965990.1 Tick phlebovirus isolate KM59
KY965991.1 Tick phlebovirus isolate KM60
KY965983.1 Tick phlebovirus isolate KM33
LC146417.1 AnLuc Phlebovirus sp. 417
LC146417.1 Phlebovirus sp. 417
LC146420.1 Phlebovirus sp. 441
KX964667.1 Phlebovirus sp. SL2-L strain Saint-Lys 2
KX964668.1 Phlebovirus sp. SL3-L strain Saint-Lys 3
LC146414.1 Phlebovirus sp. B2
LC146415.1 Phlebovirus sp. B9
LC146430.1 Phlebovirus sp. 72A
LC146432.1 Phlebovirus sp. 3A
LC146444.1 Phlebovirus sp. 82A
LC146429.1 Phlebovirus sp. 51A
LC146441.1 Phlebovirus sp. 30A
LC146436.1 Phlebovirus sp. 57A
LC146438.1 Phlebovirus sp. 10A
LC146440.1 Phlebovirus sp. 26A
LC146419.1 Phlebovirus sp. 201
LC146413.1 Phlebovirus sp. 333
LC146428.1 Phlebovirus sp. 47A
KY966010.1 Tick phlebovirus isolate ET36
KY979161.1 Tick phlebovirus isolate ED35
KY966009.1 Tick phlebovirus isolate ET33
KY966002.1 Tick phlebovirus isolate ET37
KY966006.1 Tick phlebovirus isolate ET2
MN069025. Tick phlebovirus isolate 42
MK216299.1 Phlebovirus Strandja-BG2012-GIII-strainCR
MK216298.1 Phlebovirus Strandja-BG2012-GIII-strainCQ
MN069024. Tick phlebovirus isolate 41
MK216300.1 Phlebovirus Strandja-BG2012-GIII-strainEP
KY966008.1 Tick phlebovirus isolate ET32
KY966011.1 Tick phlebovirus isolate ET31
MN069026. Tick phlebovirus isolate 43
MN069023. Tick phlebovirus isolate 40
MG764523.1 Tick phlebovirus isolate ME17
KY979168.1 Tick phlebovirus isolate MG49
LC146423.1 KarMa Phlebovirus sp. 16
KY979166.1 Tick phlebovirus isolate MG31
KY979165.1 Tick phlebovirus isolate MG22
KY979167.1 Tick phlebovirus isolate MG36
KY966007.1 Tick phlebovirus isolate ET20
KU375576.1 Phlebovirus Antigone strain Antigone2-Pella2-Greece-2013
KU375577.1 Phlebovirus Antigone strain Antigone2-Imathia1-Greece-2012
KP164566.2 Phlebovirus Antigone strain Antigone2-Imathia-Greece-2012
MN069018.Tick phlebovirus isolate 26
MN069013. Tick phlebovirus isolate 21
MN069014. Tick phlebovirus isolate 22
MN069013.Tick phlebovirus isolate 21
MN069020.Tick phlebovirus isolate 30
MN069021.Tick phlebovirus isolate 32
MN069016.Tick phlebovirus isolate 24
MN069027.Tick phlebovirus isolate 47
MN069017.Tick phlebovirus isolate 25
MN069015.Tick phlebovirus isolate 23
MN069019.Tick phlebovirus isolate 27
LC193452.1 Odaw virus strain 15AC-T35
LC193451.1 Odaw virus strain 15AC-T33
KY966005.1 Tick phlebovirus isolate ET11
KM817664.1 Bole Tick Virus 1 strain BL075
MN069022.Tick phlebovirus isolate 38
KM817672.1 Lihan Tick Virus strain LH-1
KM048311.1 American dog tick phlebovirus isolate FI6
KJ746901.1 American dog tick phlebovirus isolate ADTPV-1
KM817665.1 Changping Tick Virus 1 strain CP1-2
LC146427.1 RiPAR Phlebovirus sp. 20A
KM817684.1 Tacheng Tick Virus 2 strain TC252
MG764520.1 Tacheng Tick Virus 2 isolate MU5
KM114257.1 Silverwater virus isolate Can131
NC040494.1 Kaisodi virus isolate G14132
MG407659.1 Leuven phlebovirus isolate CH146 A490
KX184200.1 Blacklegged tick phlebovirus 1 isolate SC2
KX452150.1 Lesvos virus strain Lesvos99-Greece-2015
LC063770.1 Mukawa virus isolate MKW73
KX964671.1 Phlebovirus sp. G1-L strain Glabbeek 1
KX964669.1 Phlebovirus sp. P-L strain Prignitz
JQ924565.1 Manawa virus strain PakT 462
HM566181.1 Precarious point virus
JF838330.1 Murre virus strain Murre H
JF838327.1 Grand Arbaud virus strain Argas 27
JF838324.1 Chize virus strain Brest Ar/T2913
HM566159.1 EgAN 1825-61 virus
JQ924562.1 FinV707 virus strain FinV707
HM566191.1 Zaliv Terpenia virus
NC018136.1 SFTS virus HB29
NC024495.1 Heartland virus isolate Patient1
KC589005.1 Lone Star virus isolate TMA 1381
JX961628.1 Palma virus strain PoTi4.92
JX961616.1 Bhanja virus strain ibAr2709
JX961625.1 Forecariah virus strain DakArk4927
HQ541738.1 Gouleako virus strain A5/CI/2004
